# Isolation and characterization of 2-butoxyethanol degrading bacterial strains

**DOI:** 10.1007/s10532-020-09900-3

**Published:** 2020-04-30

**Authors:** Christine Woiski, Daniel Dobslaw, Karl-Heinrich Engesser

**Affiliations:** Department of Biological Waste Air Purification, Institute for Sanitary Engineering, Water Quality and Solid Waste Management, University of Stuttgart, Bandtaele 2, 70569 Stuttgart, Germany

**Keywords:** 2-Butoxyethanol, Degradation pathway, Glycol ether, *Pseudomonas*, *Hydrogenophaga*, *Gordonia*, *Cupriavidus*

## Abstract

A total of 11 bacterial strains capable of completely degrading 2-butoxyethanol (2-BE) were isolated from forest soil, a biotrickling filter, a bioscrubber, and activated sludge, and identified by 16S rRNA gene sequence analysis. Eight of these strains belong to the genus *Pseudomonas*; the remaining three strains are *Hydrogenophaga pseudoflava* BOE3, *Gordonia terrae* BOE5, and *Cupriavidus oxalaticus* BOE300. In addition to 2-BE, all isolated strains were able to grow on 2-ethoxyethanol and 2-propoxyethanol, ethanol, *n*-hexanol, ethyl acetate, 2-butoxyacetic acid (2-BAA), glyoxylic acid, and *n*-butanol. Apart from the only gram-positive strain isolated, BOE5, none of the strains were able to grow on the nonpolar ethers diethyl ether, di-*n*-butyl ether, *n*-butyl vinyl ether, and dibenzyl ether, as well as on 1-butoxy-2-propanol. Strains *H. pseudoflava* BOE3 and two of the isolated pseudomonads, *Pseudomonas putida* BOE100 and *P. vancouverensis* BOE200, were studied in more detail. The maximum growth rates of strains BOE3, BOE100, and BOE200 at 30 °C were 0.204 h^−1^ at 4 mM, 0.645 h^−1^ at 5 mM, and 0.395 h^−1^ at 6 mM 2-BE, respectively. 2-BAA, *n*-butanol, and butanoic acid were detected as potential metabolites during the degradation of 2-BE. These findings indicate that the degradation of 2-BE by the isolated gram-negative strains proceeds via oxidation to 2-BAA with subsequent cleavage of the ether bond yielding glyoxylate and *n*-butanol. Since *Gordonia terrae* BOE5 was the only strain able to degrade nonpolar ethers like diethyl ether, the degradation pathway of 2-BE may be different for this strain.

**Electronic supplementary material:**

The online version of this article (10.1007/s10532-020-09900-3) contains supplementary material, which is available to authorized users.

## Introduction

2-Butoxyethanol (2-BE), also called ethylene glycol monobutyl ether, is a widely used organic compound, which does not occur naturally. It is classified as a ‘High Production Volume Chemical’ (HPVC) because of its considerable production volumes of approximately 161 kt/a in the EU (2003) and 45–227 kt/a in the USA (2002) (European Chemicals Bureau 2006; OECD 2004). 2-BE is mainly used as a solvent in surface coatings, paints, and varnishes, but also in lubricants, oils, and dyes. It is found in detergents and cleaning agents, printing inks, hydrofracking liquids, oil dispersants, textiles, hair dyes, cosmetics, pharmaceutical products, agricultural chemicals, herbicides, brake fluids, de-icers, and extinguishing foams, and also serves as a starting chemical for the production of other chemicals such as butyl glycol acetate or various plasticizers (Committee on Energy and Commerce U.S. House of Representatives 2011; Gooch 2007; IARC 2006; OECD 1997; U.S. EPA 2010). 2-BE is the main component of the oil dispersant Corexit EC9527A (Kover et al. 2014). 813,000 L of Corexit EC9527A were applied to the ocean surface during the 2010 *Deepwater Horizon* oil spill (BP Gulf Science Data 2014). Consequently, 2-BE has been, and continues to be, released into the environment in large quantities through various mechanisms. Due to its high water solubility, the relatively low vapor pressure, and the low soil adsorption potential, it is predominantly found in the aqueous phase. The environmental distribution of 2-BE was predicted to be as follows: water 84.2%; air 11.4%; soil 4.19%; and sediment 0.132% (OECD 2004).

In mammals, ingested 2-BE is mainly oxidized by alcohol dehydrogenase and aldehyde dehydrogenase to 2-butoxyacetaldehyde and further to 2-butoxyacetic acid (2-BAA) (Aasmoe et al. 1998; ATSDR 1998; Bartnik et al. 1987; Boatman et al. 2014; Dean and Krenzelok 2008; Deisinger and Boatman 2004; Johanson and Johnsson 1991; Lockley et al. 2004; Rettenmeier et al. 1993; Udden and Patton 1994). 2-BAA and in some cases its glutamine and glycine conjugates were detected in urine and blood samples. Another route for the elimination of 2-BE is the conjugation with sulfate, glucuronic acid, or fatty acids (IARC 2006; Kaphalia et al. 1996). To a much lesser extent, dealkylation of 2-BE by cytochrome P450 2E1 (CYP 2E1) was observed. This reaction leads to the formation of butyraldehyde and ethylene glycol, which is further oxidized to oxalic acid (Rambourg-Schepens et al. 1988).

The degradation pathway for 2-BE in bacteria, however, has not yet been studied in detail. 2-BE is an aliphatic ether comprising a nonpolar alkyl residue on one side and a polar ethylene glycol residue on the other side. Primary degradation can occur by attacking either group. Different pathways for the aerobic degradation of ethylene glycol ethers (EGEs) like polyethylene glycol (PEG) or linear primary fatty alcohol ethoxylates (LAEs) on the one hand and linear and cyclic alkyl ethers (AEs) on the other hand have been proposed. They can provide information on the possible degradation route of 2-BE. The focus of this work is the 2-BE degradation under aerobic conditions. Therefore, anaerobic pathways were not considered.

**Fig. 1:**
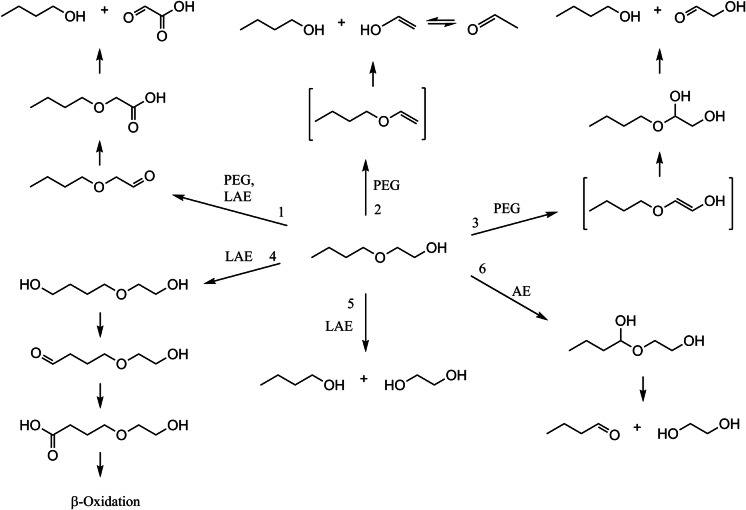
Possible degradation pathways for 2-BE, according to the literature. Details can be found in the introduction.

Three different pathways for the degradation of PEG have been postulated. Most frequently, oxidation of the hydroxyl group to a carboxyl group was observed, followed by the cleavage of the ether bond yielding glyoxylate and PEG shortened by one glycol unit. Bacterial strains degrading PEG in this manner include *Acinetobacter* SC 25 and *Pseudomonas* KW 8 (degrading up to PEG 400), *Flavobacterium* BT 1 (up to PEG 1500) (Watson and Jones 1977), the gram-negative strains PG1, PG3, PG5, and PG6 (up to PEG 400) (Hosoya et al. 1978), *Pseudonocardia* sp. strain K1 (up to PEG 8000) (Kohlweyer et al. 2000; Yamashita et al. 2004a), *Sphingomonas* sp. N6 (up to PEG 20,000) (Kawai and Enokibara 1996), *Sphingopyxis macrogoltabida* (formerly *Flavobacterium* and *Sphingomonas macrogoltabidus*) strains 103 and 203 (up to PEG 4000) (Kawai and Yamanaka 1989; Takeuchi et al. 1993,2001; Yamanaka and Kawai 1989), and the mixed culture E-1 consisting of *Sphingopyxis terrae* (formerly *Flavobacterium* and *Sphingomonas terrae*) and *Rhizobium* sp. (formerly *Pseudomonas*) (up to PEG 20,000) (Kawai and Yamanaka 1986, 1989; Takeuchi et al. 2001). Two ether-cleaving enzymes have been identified, a PEG carboxylate dehydrogenase (PCDH) from *S. terrae* and *S. macrogoltabida* strains 103 and 203 (Somyoonsap et al. 2008; Tani et al. 2007), and a diglycolic acid dehydrogenase (DGADH) from *Pseudonocardia* sp. K1 (Yamashita et al. 2004a). The degradation of PEG by *Pseudomonas stutzeri* JA1001 (up to PEG 13,500) also occurs via oxidation of the alcohol to the corresponding acid with subsequent elimination of glyoxylate (Obradors and Aguilar 1991). However, it was suggested that a single enzyme catalyzes all three reaction steps at once. The (hypothetic) degradation of 2-BE according to this pathway is depicted in Fig. 1, route 1.

Another degradation route was proposed by Pearce and Heydeman (1980) for *Acinetobacter* strain S8. First, a (hypothetical) vinyl ether intermediate is formed by the elimination of water. In the next step, water is added and the ether is cleaved yielding PEG shortened by one glycol unit, and vinyl alcohol, which is in tautomeric balance with acetaldehyde (Fig. 1, route 2).

Pathway no. 3 was proposed by Thélu et al. (1980) for the degradation of PEG 400 by *Pseudomonas* P400. After the formation of an enol ether, a hemiacetal is produced by water addition. The ether is cleaved thereafter, either spontaneously or by formation of an ester and subsequent hydrolysis (Fig. 1, route 3).

The breakdown of LAEs, which are used as nonionic surfactants, was examined by several groups and, again, three different primary attacks were observed (Schröder 2001). Degradation mechanisms were determined by the detection of metabolites. In some cases, intermediates have been found within one culture indicating that at least two of these pathways occur in parallel.

First, the alkyl chain is ω- and subsequently β-oxidized (Fig. 1, route 4), for example by a mixed culture including *Pseudomonas putida* TSh-18, *Pseudomonas putida* TP-19, and *Pseudomonas* sp. OS-22 (Panchenko and Turkovskaya 2000) and by microorganisms within activated sludge (Patterson et al. 1967, 1970; Steber and Wierich 1985).

Second, the PEG moiety is attacked by oxidation of the hydroxyl group and oxidative shortening of the polyether chain, according to the first PEG degradation pathway mentioned. Strains *Pseudomonas* sp. RW1 (Corti et al. 1998), *Enterobacter* strain Z3 (Zembrzuska et al. 2016), and again the mixed culture including *Pseudomonas putida* TSh-18, *Pseudomonas putida* TP-19, and *Pseudomonas* sp. OS-22 (Panchenko and Turkovskaya 2000) are able to degrade LAEs in this manner (Fig. 1, route 1).

Third, central fission of the ether bond with the formation of a fatty alcohol and the PEG moiety took place in pure cultures of *Pseudomonas* sp. strain SC25A (Tidswell et al. 1996), *Enterobacter* strains Z2 and Z3, *Citrobacter freundii* strain Z4, *Stenotrophomonas* strain Z5 (Budnik et al. 2016; Zembrzuska et al. 2016), a pseudomonad (Ichikawa et al. 1978), and also in activated sludge (Patterson et al. 1970; Sparham et al. 2008; Steber and Wierich 1985; Szymanski et al. 2002a, b; Tobin et al. 1976) (Fig. 1, route 5). Details about the ether cleavage are not known, although this mechanism seems to be widespread.

The degradation of nonpolar AE starts by hydroxylation of the c-atom adjacent to the ether bond, forming an unstable hemiacetal (Fig. 1, route 6). Different enzymes catalyzing this reaction have been described. Toluene 2-monooxygenase from *Burkholderia cepacia* G4/PR1 oxidizes diethyl ether, butyl methyl ether, and 2-chloroethyl ethyl ether (Hur et al. 1997). The cytochrome P450 monooxygenase Eth from *Aquincola tertiaricarbonis* L108 catalyzes the *O*-dealkylation of diethyl ether as well as of methyl *tert*-butyl ether (MTBE) (Schuster et al. 2013) and ammonia monooxygenase from *Nitrosomonas europaea* transforms dimethyl ether and diethyl ether (Hyman 1999). The same reaction type is assumed to occur in various strains of the genus *Rhodococcus* (Bock et al. 1996; Kim et al. 2007; Kim and Engesser 2004; Moreno-Horn et al. 2005; Tajima et al. 2012). Tetrahydrofuran (THF) degrading *Pseudonocardia* sp. K1, *Pseudonocardia* sp. strain ENV478, and *Rhodococcus aetherivorans* strain M8, as well as 1,4-dioxane degrading *P. dioxanivorans* CB1190, are also capable of growing on diethyl ether (Kohlweyer et al. 2000; Parales et al. 1994; Tajima et al. 2012; Vainberg et al. 2006). Different soluble di-iron monooxygenases (SDIMO) are involved in THF or 1,4-dioxane degradation, namely THF monooxygenase Thm, 1,4-dioxane monooxygenase Dxm, and propane monooxygenase Prm (Inoue et al. 2016; Thiemer et al. 2003). *Pseudonocardia* sp. strain ENV478 transformed bis(2-chloroethyl) ether only after growing on propane or THF (McClay et al. 2007). Therefore, it is very likely that SDIMOs act on diethyl ether as well.

As already mentioned, *Pseudonocardia* sp. K1 degrades PEG besides THF (Yamashita et al. 2004a). THF is cleaved by Thm (Inoue et al. 2016). PEG on the other hand is first oxidized to PEG carboxylate, and then cleaved by superoxide dismutase-like DGADH (Yamashita et al. 2004b). This strain thus degrades PEG and THF by two completely different pathways.

In summary, 6 different primary attacks were considered for the degradation of 2-BE analogous to the degradation of PEG, LAE and AE, which are presented in Fig. 1. The aim of this study was to isolate and characterize bacterial strains able to degrade 2-BE and to gain initial information about the degradation pathway.

## Materials and methods

### Culture media

The strains were cultivated in mineral salt medium (MM) consisting of (per liter) KH_2_PO_4_ 1 g; Na_2_HPO_4_·2H_2_O 3.5 g; (NH_4_)_2_SO_4_ 1 g; MgSO_4_·7H_2_O 200 mg; Ca(NO_3_)_2_·4H_2_O 50 mg; Fe(III)NH_4_-citrate 10 mg; H_3_BO_3_ 0.3 mg; CoCl_2_·6H_2_O 0.2 mg; ZnSO_4_·7 H_2_O 0.1 mg; MnCl_2_·4H_2_O 30 µg; NaMoO_4_·2H_2_O 30 µg; NiCl_2_·6H_2_O 20 µg; CuCl_2_·2H_2_O 10 µg supplemented with a carbon source. MMB medium consists of MM with addition of 5 mM 2-BE. Lysogenic broth (LB) contained (per liter) 10 g tryptone, 5 g yeast extract, and 10 g NaCl. For solid medium, 1.5% agar was added.

### Isolation and identification of 2-BE degrading bacterial strains

Different samples were used as inocula for enrichment cultures: forest soil, samples from a biotrickling filter (both Stuttgart, Germany), samples from a bioscrubber (Rastatt, Germany), and activated sludge (Stuttgart and Karlsruhe, Germany). The biotrickling filter was used to clean air contaminated with 2-BE (Dobslaw et al. 2018). The bioscrubber removed VOCs in varying concentrations including butyl acetate, ethyl acetate, methyl ethyl ketone (MEK), acetone, dichloromethane (DCM), ethanol, methanol, isopropanol, THF, hexane, heptane, ethylbenzene, 2-BE, and others (Dobslaw et al. 2010). Approximately 2 g of each sample were transferred into 250 ml flasks containing 50 ml MM medium and 3 mM 2-BE as the sole source of carbon. The flasks were incubated at 30 °C on a rotary shaker (150 rpm) for one week. About 5 ml of these cultures were transferred into flasks with fresh medium and incubated under the same conditions. After another transfer, dilutions of the cultures were plated on solid MMB and incubated at 30 °C. Single colonies were spread on fresh plates to obtain pure strains.

BOX PCR fingerprinting using primer BOXA1R (CTACGGCAAGGCGACGCTGACG) showed whether the isolated strains differ from each other (Martin et al. 1992; Versalovic et al. 1994). The strains were identified by amplification of parts of the 16S rRNA gene using Primers 27F (AGAGTTTGATCMTGGCTCAG) and 1492R (TACGGYTACCTTGTTACGACTT) (Weisburg et al. 1991). The PCR products were purified using DNA Clean & Concentrator-5 Kit (Zymo Research Europe GmbH, Freiburg, Germany). Sanger sequencing of the purified amplicons was performed by Microsynth Seqlab (Goettingen, Germany). To identify the strains, 16S rRNA gene sequences were analyzed using the BLAST program (Altschul et al. 1990). The sequences were deposited in GenBank under accession numbers MH580159 and MH580208–MH580217 for strains BOE1, BOE2, BOE3, BOE4, BOE5, BOE6, BOE7, BOE10, BOE100, BOE200, and BOE300, respectively.

A phylogenetic tree based on the 16S rRNA gene sequences of the isolated strains and closely related type strains was constructed by the Neighbor-Joining method (Saitou and Nei 1987) using MEGA7 (Kumar et al. 2016). The evolutionary distances were computed using the Maximum Composite Likelihood method (Tamura et al. 2004).

### Substrate utilization

Different substrates were used for growth experiments to obtain information on the substrate versatility of the isolated strains and the possible degradation pathway of 2-BE. MM medium was inoculated with cells of the respective strain to obtain an optical density at 546 nm (OD_546_) of at least 0.1. Cell densities were measured using Pharmacia LKB Biotechnology Ultrospec III UV/Visible Spectrophotometer. 3 mM of the following compounds were added as sole source of carbon: 2-BAA, glyoxylic acid, *n*-butanol, ethanol, *n*-hexanol, ethyl acetate, isopropanol, acetone, MEK, diethyl ether, di-*n*-butyl ether, *n*-butyl vinyl ether, dibenzyl ether, 2-ethoxyethanol, PEG 200, 2-propoxyethanol, 1-butoxy-2-propanol, 2-phenoxyethanol, hexane, cyclohexane, benzoic acid, styrene, and toluene. The cultures were incubated at 30 °C on a rotary shaker and cell densities were measured after one week.

### Growth experiments

Liquid MMB was inoculated with cells of the strains *H. pseudoflava* BOE3, *P. putida* BOE100, or *P. vancouverensis* BOE200 grown on MM agar plates containing 7.6 mM 2-BE. Cultures were incubated over night at 30 °C on a rotary shaker (150 rpm). The next day, cultures were diluted with fresh medium to obtain an OD_546_ of 0.06–0.1. 2-BE was added at different concentrations, and the OD_546_ was measured periodically. The experiments were conducted in triplicates.

### Salt tolerance

3.5% (w/v) sodium chloride was added to liquid MMB medium, approximately corresponding to the salinity of seawater, and growth was monitored to analyze the salt tolerance of strains BOE3, BOE100, and BOE200.

### Antibiotic resistance

Strains BOE100 and BOE200 were spread on LB plates and strain BOE3 was spread on MMB plates, because it does not grow on LB plates. Sterile filter disks were placed on top. The following masses of different antibiotics were applied to the disks: chloramphenicol (Cm) 64 µg, tetracycline 12 µg, kanamycin 50 µg, ampicillin 50 µg, penicillin 50 µg, streptomycin 50 µg, nalidixic acid 50 µg. The plates were incubated at 30 °C for 1 day (BOE100), 2 days (BOE200), or 5 days (BOE3), as growth rates of the three strains examined varied. Thereafter, the size of the zone of inhibition was measured.

### Metabolite detection

Cultures were grown over night in MMB medium. The next day, 10 mM 2-BE were added and the cultures were incubated for another 3 h. Cm was added to stop protein synthesis and the cultures were incubated overnight. The medium was centrifuged at 5000×*g* for 15 min at room temperature to remove cells. The supernatant was acidified with phosphoric acid to pH 2 and extracted with an equal volume of DCM for 3 min. Extracts were dried over anhydrous sodium sulfate and analyzed by GC–MS [Agilent Technologies; 7890A GC system, 5975C VL MSD detector, 7683B injector, VF-5 ms column (60 m × 0,25 mm × 0,25 µm)]. The temperature program was as follows: hold for 1 min at 40 °C, increase to 200 °C at 10 °C min^−1^. 1 µl was injected and the sample was analyzed in split mode (1:10). Helium was used as carrier gas at a flow rate of 0.927 ml min^−1^. The goal was to detect potential metabolites qualitatively, not quantitatively. Therefore, the creation of calibration curves was omitted.

## Results

### Isolation and identification of 2-BE degrading bacterial strains

**Fig. 2:**
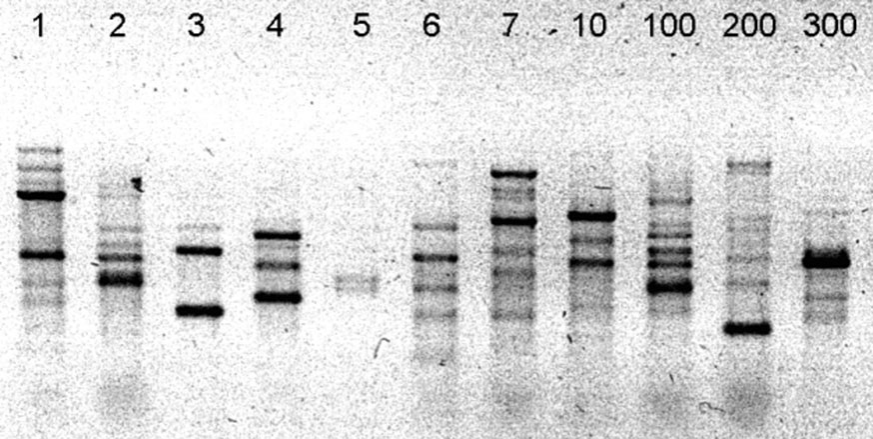
BOX PCR fingerprinting showing that the isolated strains differ genetically from each other. 1 BOE1, 2 BOE2, 3 BOE3, 4 BOE4, 5 BOE5, 6 BOE6, 7 BOE7, 10 BOE10, 100 BOE100, 200 BOE200, 300 BOE300

**Fig. 3:**
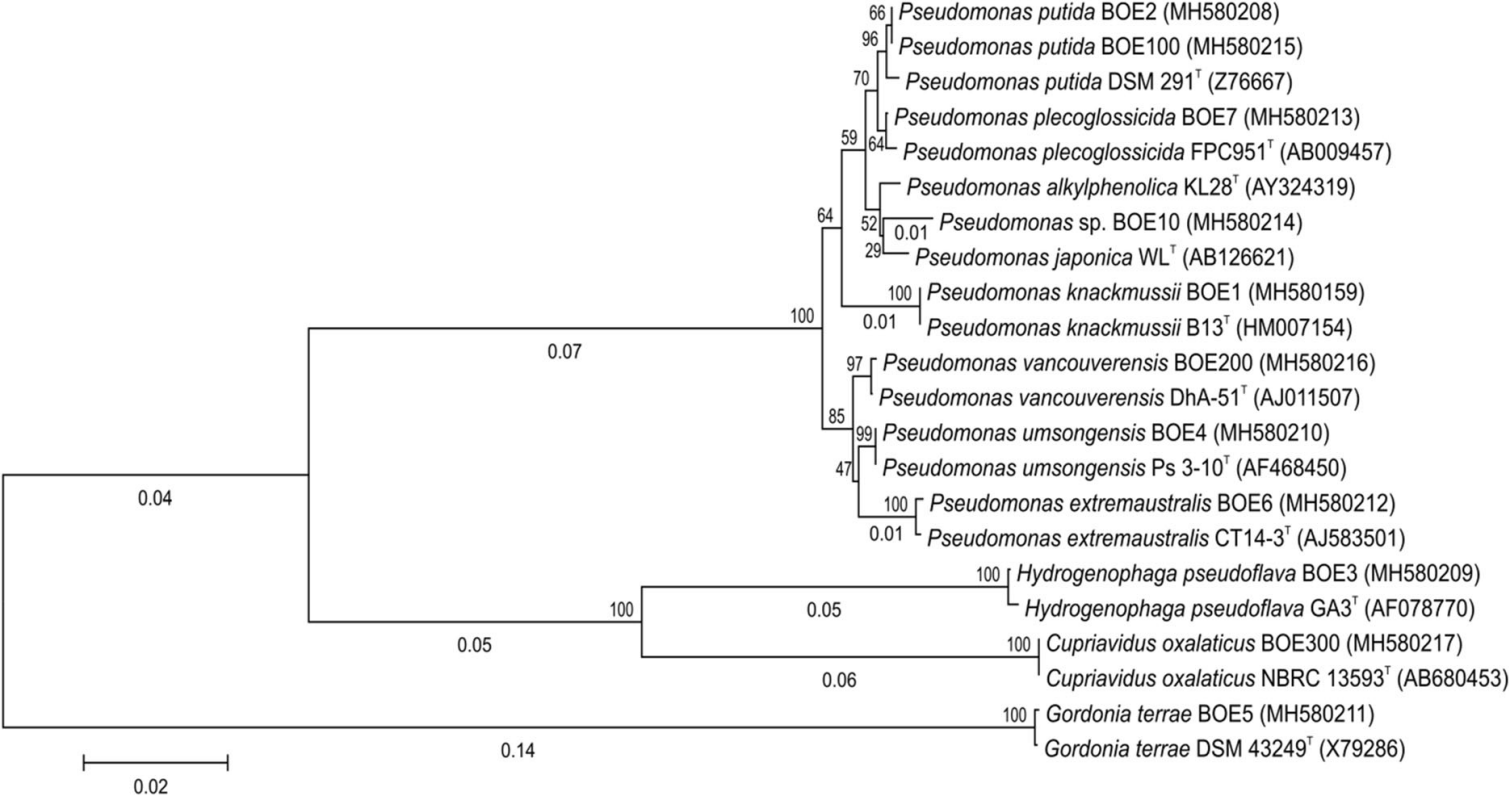
Phylogenetic tree constructed by the Neighbor-Joining method (Saitou and Nei 1987) based on the 16S rRNA gene sequences of the isolated strains and closely related type strains (T). GenBank accession numbers are presented in parentheses. The percentage of replicate trees in which the associated taxa clustered together in the bootstrap test (1000 replicates) is shown next to the nodes. The tree is drawn to scale, with branch lengths (below the branches) in the same units as those of the evolutionary distances used to infer the phylogenetic tree. The evolutionary distances were computed using the Maximum Composite Likelihood method (Tamura et al. 2004) and are in the units of the number of base substitutions per site. The scale bar represents 0.02 substitutions per nucleotide position. There were a total of 1265 positions in the final dataset. Evolutionary analyses were conducted in MEGA7 (Kumar et al. 2016)

In total, 11 distinct 2-BE degrading bacterial strains were isolated. As shown in Fig. 2, the BOX PCR fingerprint of each isolated strain was unique, which means the strains differ from each other. All strains are catalase-positive and oxidase-positive. On the basis of 16S rRNA gene sequence analysis, they were identified as *Pseudomonas knackmussii* BOE1, *Pseudomonas putida* BOE2, *Hydrogenophaga pseudoflava* BOE3, *Pseudomonas umsongensis* BOE4, *Gordonia terrae* BOE5, *Pseudomonas extremaustralis* BOE6, *Pseudomonas plecoglossicida* BOE7, *Pseudomonas* sp. BOE10, *Pseudomonas putida* BOE100, *Pseudomonas vancouverensis* BOE200, and *Cupriavidus oxalaticus* BOE300. Table 1 shows the origin of the isolated strains and the GenBank accession numbers of their corresponding 16S rRNA gene sequences. The phylogenetic tree constructed on the basis of the 16S rRNA gene sequences of these strains and closely related type strains is shown in Fig. 3.

**Table 1:** Isolated 2-BE degrading strains, their origin, and the GenBank accession no. of their 16S rRNA sequence

Bacterial strain	Gram	Inoculum	GenBank accession no
*Pseudomonas knackmussii* BOE1	Gram-negative	Forest soil, Stuttgart (Germany)	MH580159
*Pseudomonas putida* BOE2	Gram-negative	Forest soil, Stuttgart (Germany)	MH580208
*Hydrogenophaga pseudoflava* BOE3	Gram-negative	Forest soil, Stuttgart (Germany)	MH580209
*Pseudomonas umsongensis* BOE4	Gram-negative	Forest soil, Stuttgart (Germany)	MH580210
*Gordonia terrae* BOE5	Gram-positive	Biotrickling filter, Stuttgart (Germany)	MH580211
*Pseudomonas extremaustralis* BOE6	Gram-negative	Biotrickling filter, Stuttgart (Germany)	MH580212
*Pseudomonas plecoglossicida* BOE7	Gram-negative	Activated sludge, Karlsruhe (Germany)	MH580213
*Pseudomonas* sp. BOE10	Gram-negative	Activated sludge, Karlsruhe (Germany)	MH580214
*Pseudomonas putida* BOE100	Gram-negative	Bioscrubber, Rastatt (Germany)	MH580215
*Pseudomonas vancouverensis* BOE200	Gram-negative	Bioscrubber, Rastatt (Germany)	MH580216
*Cupriavidus oxalaticus* BOE300	Gram-negative	Activated sludge, Stuttgart (Germany)	MH580217

### Substrate utilization

Substrate utilization of the BOE strains is summarized in Table 2. All strains were able to grow on 2-BE and its possible degradation metabolites 2-BAA, glyoxylic acid, and *n*-butanol. Additionally, the alcohols ethanol and *n*-hexanol, the ester ethyl acetate, and the EGEs 2-ethoxyethanol and 2-propoxyethanol served as growth substrates. Unlike the other strains, BOE3 and BOE100 could grow on the further tested EGE PEG 200. Remarkably, only BOE5 grew on the nonpolar ethers diethyl ether, di-*n*-butyl ether, *n*-butyl vinyl ether, and dibenzyl ether, as well as on the propylene glycol ether 1-butoxy-2-propanol. 2-phenoxyethanol was used as growth substrate by strains BOE2 and BOE4. Isopropanol and acetone, the C3 substrates tested, served strains BOE5 and BOE300 as growth substrates. Strains BOE5, BOE6, BOE200, and BOE300 were able to grow on MEK. BOE2, BOE4, BOE5, BOE7, BOE100, BOE200, and BOE300 grew on benzoic acid. None of the strains were able to use either the (cyclo-)alkanes hexane and cyclohexane or the nonpolar aromatic compounds styrene and toluene as growth substrates.

**Table 2:** Substrate utilization of the BOE strains

Substrate	BOE 1	BOE 2	BOE 3	BOE 4	BOE 5	BOE 6	BOE 7	BOE 10	BOE 100	BOE 200	BOE 300
2-BE	+	+	+	+	+	+	+	+	+	+	+
2-BAA	+	+	+	+	+	+	+	+	+	+	+
Glyoxylic acid	+	+	+	+	+	+	+	+	+	+	+
*n*-Butanol	+	+	+	+	+	+	+	+	+	+	+
Ethanol	+	+	+	+	+	+	+	+	+	+	+
*n*-Hexanol	+	+	+	+	+	+	+	+	+	+	+
Ethyl acetate	+	+	+	+	+	+	+	+	+	+	+
Isopropanol	−	−	−	−	+	−	−	−	−	−	+
Acetone	−	−	−	−	+	−	−	−	−	−	+
MEK	−	−	−	−	+	+	−	−	−	+	+
Diethyl ether	−	−	−	−	+	−	−	−	−	−	−
Di-*n*-butyl ether	−	−	−	−	+	−	−	−	−	−	−
*n*-Butyl vinyl ether	−	−	−	−	+	−	−	−	−	−	−
Dibenzyl ether	−	−	−	−	+	−	−	−	−	−	−
2-Ethoxyethanol	+	+	+	+	+	+	+	+	+	+	+
PEG 200	−	−	+	−	−	−	−	−	+	−	−
2-Propoxyethanol	+	+	+	+	+	+	+	+	+	+	+
1-Butoxy-2-propanol	−	−	−	−	+	−	−	−	−	−	−
2-Phenoxyethanol	−	+	−	+	−	−	−	−	−	−	−
Hexane	−	−	−	−	−	−	−	−	−	−	−
Cyclohexane	−	−	−	−	−	−	−	−	−	−	−
Benzoic acid	−	+	−	+	+	−	+	−	+	+	+
Styrene	−	−	−	−	−	−	−	−	−	−	−
Toluene	−	−	−	−	−	−	−	−	−	−	−

Liquid MM medium was inoculated with the respective strain, 3 mM substrate was added and the OD_546_ was measured after one week.+, OD_546_ increased by ≥ 0.2; −, OD_546_ increased by < 0.2 or decreased

### Growth experiments

**Fig. 4:**
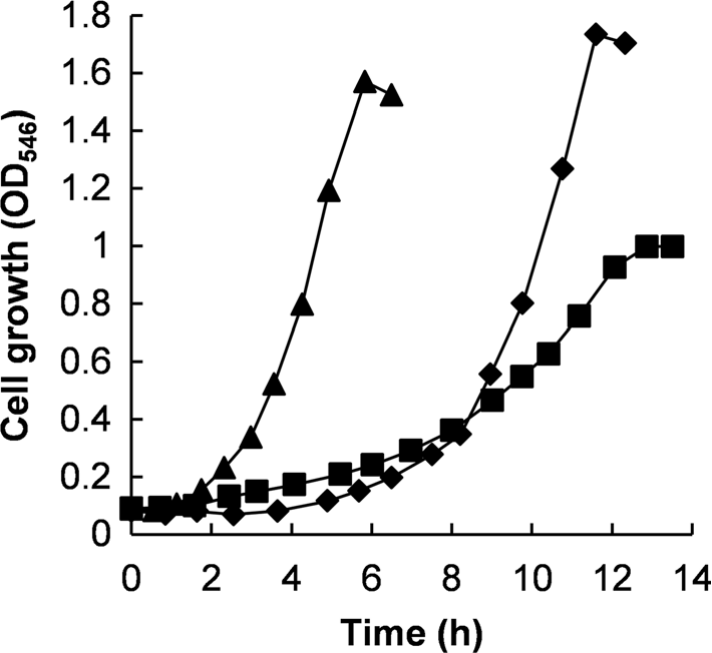
Maximum cell growth of strains BOE3 at 4 mM 2-BE (squares), BOE100 at 5 mM 2-BE (triangles), and BOE200 at 6 mM 2-BE (diamonds). At these concentrations, the maximum growth rates were obtained among all concentrations tested

BOE3 was able to grow with 4 mM, 6 mM, 8 mM, and 10 mM 2-BE with slightly decreasing growth rates during exponential phase of 0.204 h^−1^, 0.198 h^−1^, 0.194 h^−1^, and 0.193 h^−1^, respectively. In comparison, BOE100 was able to grow faster and tolerated higher 2-BE concentration. The growth rates for 2-BE concentrations of 5 mM, 7.5 mM, 10 mM, 12.5 mM, and 15 mM were 0.645 h^−1^, 0.602 h^−1^, 0.565 h^−1^, 0.538 h^−1^, and 0.514 h^−1^, respectively. BOE200 grew with 2-BE concentrations of 4 mM, 6 mM, 8 mM, and 10 mM at growth rates of 0.383 h^−1^, 0.395 h^−1^, 0.363 h^−1^, and 0.362 h^−1^. Accordingly, the highest growth rate (0.645 h^−1^) was achieved by BOE100 at a 2-BE concentration of 5 mM. Figure 4 shows the growth curves for strains BOE3, BOE100, and BOE200 for 2-BE concentrations of 4 mM, 5 mM, and 6 mM, respectively, since these were the concentrations leading to the highest growth rates for each strain. BOE200 had a lag phase of about 4 h, whereas growth occurred immediately for strains BOE3 and BOE100. Within the 2-BE concentration range tested, the consumption of 1 mM 2-BE resulted in an average increase of OD_546_ of 0.227 for BOE3, 0.29 for BOE100, and 0.273 for BOE200.

**Fig. 5:**
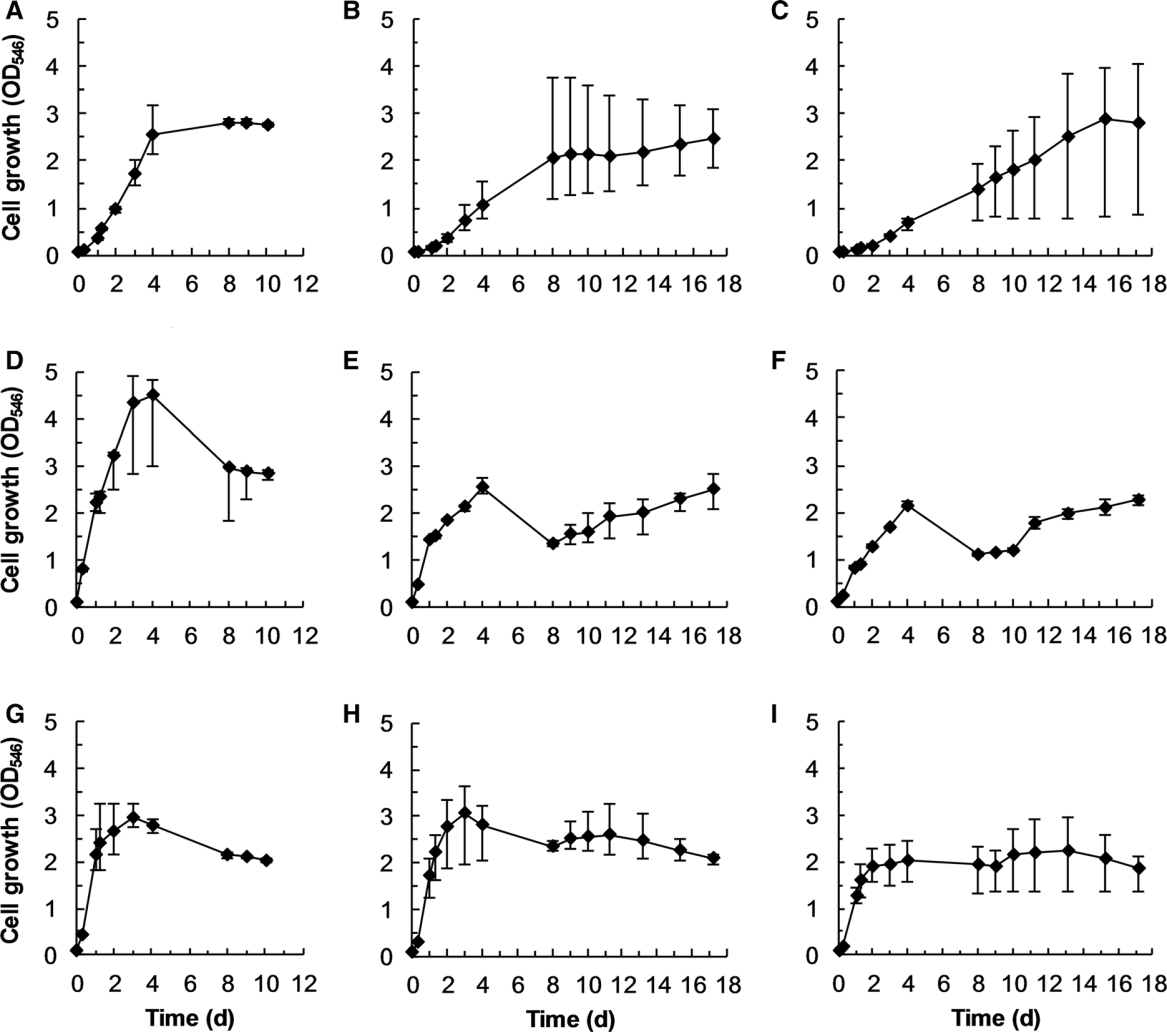
Growth experiments were conducted in triplicate for strains BOE3, BOE100, and BOE200 at different 2-BE concentrations. A BOE3, 20 mM; B BOE3, 30 mM; C BOE3, 40 mM; D BOE100, 30 mM; E BOE100, 40 mM; F BOE100, 50 mM; G BOE200, 20 mM; H BOE200, 30 mM; I BOE200, 40 mM

Growth of strain BOE3 with 20 mM, 30 mM, and 40 mM 2-BE is depicted in Fig. 5, panels A–C. 20 mM 2-BE were consumed completely within 4 days and a maximum OD_546_ of about 3 was reached (panel A). At day 1, growth rates were 0.071–0.077 h^−1^. On days 1–4, they slowed down to about 0.02–0.027 h^−1^. Growth with 30 mM and 40 mM 2-BE was considerably slower and the three cultures for each concentration varied strongly in their pace of growth (panels B and C). The growth rates were 0.032–0.04 h^−1^ on days 1–3, 0.005–0.01 h^−1^ on days 3–8, and 0–0.003 h^−1^ on days 8–17 at 30 mM 2-BE, and 0.021–0.026 h^−1^ on days 1–4 and 0.001–0.007 h^−1^ on days 8–17 at 40 mM 2-BE. At these concentrations, growth was considerably inhibited and not reproducible and the OD_546_ did not exceed the value of 4.

Panels D–F of Fig. 5 show the growth of strain BOE100 with 30 mM, 40 mM, and 50 mM 2-BE. 30 mM 2-BE were degraded within 4–10 days (panel D). Growth rates were calculated to be ca. 0.113–0.124 h^−1^ on day 1 and 0.008–0.019 h^−1^ on days 1–3. The maximum OD_546_ was about 4.5. At higher concentrations, growth rates declined substantially (day 1, 0.087–0.104 h^−1^; days 1–4, 0.008–0.012 h^−1^, days 8–18, 0.002–0.004 h^−1^) and the highest OD_546_ was 3 (panels E and F). 2-BAA was detected in the medium at day 4 and the pH was about 6. After 18 days, 2-BAA was completely removed and the pH increased to 7. Growth between days 8 and 18 was limited, the OD_546_ increased only to a comparatively small extent from 1.3 to 2.5 with 40 mM 2-BE, and from 1.1 to 2.3 with 50 mM 2-BE.

Growth of strain BOE200 with 20 mM, 30 mM and 40 mM 2-BE is shown in panels G–I. After 3 days, 20 mM 2-BE were consumed and the OD_546_ was about 3. Growth rates were 0.118–0.138 h^−1^ on day 1 and about 0.01 h^−1^ on days 1–3. Growth rates at 30 mM 2-BE were similar to those at 20 mM until day 3 (day 1, 0.109–0.13 h^−1^; days 1–3, 0.004–0.009 h^−1^), although 2-BE was not degraded completely during this time (panel H). After 4 days, 2-BAA accumulated and pH dropped to approximately 6, inhibiting further growth. This was already observed with BOE100. Panel I shows that 40 mM 2-BE led up to an OD_546_ of about 2 within 4 days with growth rates of 0.107–0.118 on day 1 and 0.003–0.004 on days 1–4. This was the highest OD_546_ reached and continued growth was hindered. Again, 2-BAA was detected in the medium and the pH was about 6. 2-BE and 2-BAA were completely removed after 18 days at both 40 mM and 50 mM initial concentrations, but slowly and, as already stated, without any additional growth.

### Characterization of strains *H. pseudoflava* BOE3, *P. putida* BOE100, and *P. vancouverensis* BOE200

Strains BOE3, BOE100, and BOE200 are sensitive to Cm, tetracycline, kanamycin, streptomycin, and nalidixic acid, as shown in Table 3. Whereas strain BOE3 is susceptible to ampicillin and penicillin, the pseudomonads BOE100 and BOE200 are resistant to those two beta-lactam antibiotics.

**Table 3:** Antibiotic resistance of strains BOE3, BOE100, and BOE200

Antibiotic	Mass	Inhibition zone size [mm]		
	µg	BOE3	BOE100	BOE200
Chloramphenicol	64	32	12	17
Tetracycline	12	38	19	25
Kanamycin	50	60	22	24
Ampicillin	50	38	0	0
Penicillin	50	40	0	0
Streptomycin	50	58	16	20
Nalidixic acid	50	60	14	22

**Fig. 6:**
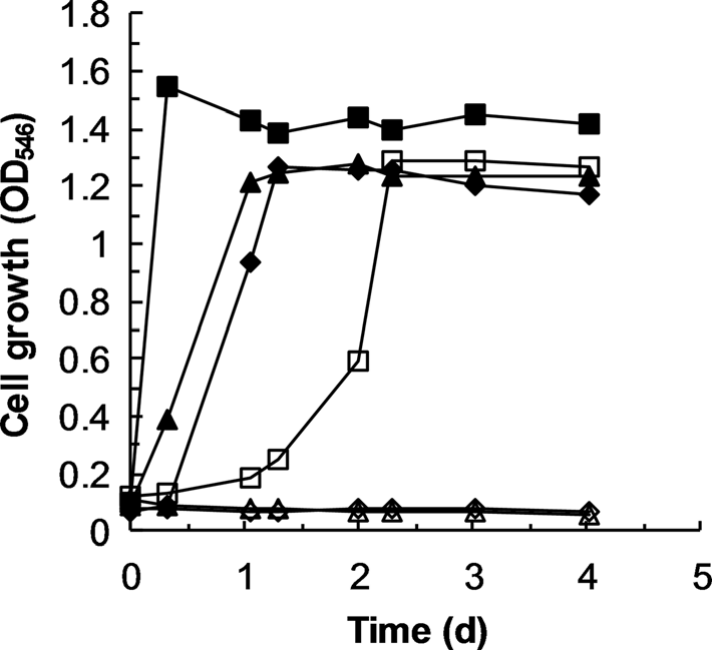
Growth of BOE3, BOE100, and BOE200 in MMB with and without the addition of 3.5% NaCl. Filled diamonds: BOE3, 0% NaCl; open diamonds: BOE3, 3.5% NaCl; filled squares: BOE100, 0% NaCl; open squares: BOE100, 3.5% NaCl; filled triangles: BOE200, 0% NaCl; open triangles: BOE200, 3.5% NaCl

As already mentioned in the introduction, 2-BE is the main component of Corexit EC9527A and 813,000 L of this dispersant were applied to the ocean surface during the 2010 *Deepwater Horizon* oil spill. Therefore, strains BOE3, BOE100 and BOE200 were studied for their ability to degrade 2-BE under marine conditions. Only strain BOE100 was able to grow in MMB containing 3.5% NaCl (Fig. 6). Growth started after an adaption period of one day and the growth rate of 0.065 h^−1^ was considerably lower than in MMB without NaCl. Strains BOE3 and BOE200 showed no growth under these conditions.

### Metabolite detection

**Fig. 7:**
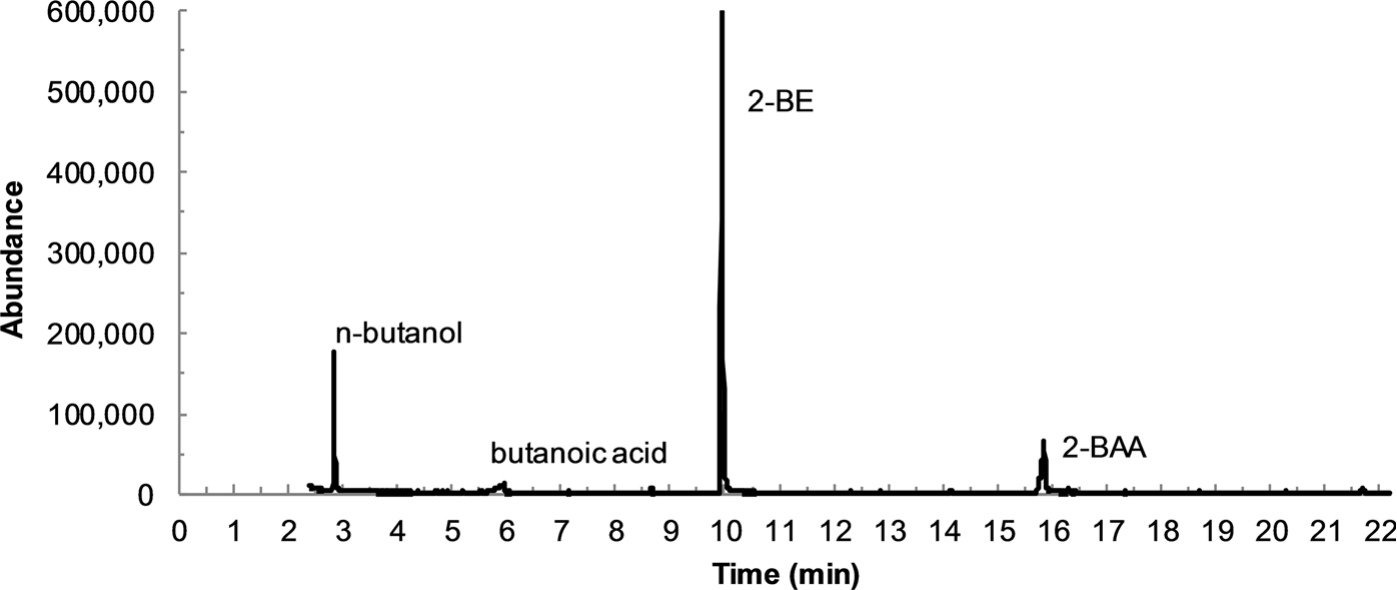
GC–MS chromatogram of a DCM extract of a BOE100 culture grown in MMB after addition of Cm and incubation for another day showing the metabolites *n*-butanol, butanoic acid, and BAA as well as the substrate 2-BE

BOE100 was grown in liquid MMB and Cm was added during exponential growth to stop protein synthesis. 10 mM 2-BE were added and the culture was incubated for another day. It was then centrifuged and the supernatant was extracted with DCM and analyzed by GC–MS. Figure 7 shows the chromatogram. In addition to the substrate 2-BE, 2-BAA, *n*-butanol, and butanoic acid were detected as potential intermediates of the 2-BE degradation pathway.

## Discussion

11 bacterial strains capable of degrading 2-BE were isolated from soil, activated sludge from different waste water treatment plants, a biotrickling filter, and a bioscrubber, and identified as *Pseudomonas knackmussii* BOE1, *Pseudomonas putida* BOE2, *Hydrogenophaga pseudoflava* BOE3, *Pseudomonas umsongensis* BOE4, *Gordonia terrae* BOE5, *Pseudomonas extremaustralis* BOE6, *Pseudomonas plecoglossicida* BOE7, *Pseudomonas* sp. BOE10, *Pseudomonas putida* BOE100, *Pseudomonas vancouverensis* BOE200, and *Cupriavidus oxalaticus* BOE300. Among these strains, only *G. terrae* BOE5 is gram-positive, and 8 out of the 10 gram-negative strains are pseudomonads. All strains were catalase-positive and oxidase-positive. Isolated from very different sources and locations, P*seudomonas* seemed to be the dominant genus harboring the 2-BE degradation capacity in the environment.

In the past, many *Pseudomonas* strains have been isolated degrading a broad variety of organic molecules including alkanes (Rojo 2009; van Beilen and Funhoff 2007; Wang et al. 2017), aromatic compounds (Arias et al. 2008; Jindrová et al. 2002; Mooney et al. 2006; Peng et al. 2008), nonpolar ethers like THF (Chen et al. 2010), 3- and 4-phenoxybenzoate (Dehmel et al. 1995; Engesser et al. 1990; Topp and Akhtar 1991; Wittich et al. 1990), dibenzo-*p*-dioxin, dibenzofuran, 1- and 2-monochlorodibenzo-*p*-dioxin (Hong et al. 2004), polymers like polyacrylate, polyhydroxyalkanoates, and polyvinyl alcohol (Kawai 2010), and more (Palleroni et al. 2010; Wackett 2003). Pseudomonads have also been described degrading PEG 400 (*Pseudomonas* sp. KW 8) (Watson and Jones 1977) or even PEG 13,500 (*Pseudomonas stutzeri* JA1001) (Obradors and Aguilar 1991). A PEG dehydrogenase was identified in the periplasm of strain JA1001. Activity of that enzyme was assayed by measuring the change in absorbance at 600 nm after the addition of 2,6-dichlorophenol-indophenol. PEG, diethylene glycol, and diglycolic acid were oxidized. Thus, the authors claim that this enzyme both oxidizes PEG and cleaves the ether bond. No further details are known about this enzyme such as molecular weight or amino acid sequence. Accordingly, no ether-cleaving genes have been identified in this genus and no degradation kinetics have been published.

Yet, not every strain of the genus *Pseudomonas* is able to degrade 2-BE. We tested several pseudomonads from our strain collection for growth on 2-BE, including *P. putida* KT2440 (Bagdasarian et al. 1981), *P. putida* F1 (Zylstra et al. 1988), *P. fluorescens* DSM 56106, *P. veronii* MEK700 (Onaca et al. 2007), *P. vancouverensis* NCIMB 9816 (Kurkela et al. 1988), *P. abietaniphila* ATCC 17,483 (Barnsley 1976), and strains isolated from Annika Buchwald of our working group, *Pseudomonas* sp. BAL210 and *Pseudomonas* sp. BAL220. None of these strains were able to grow on 2-BE. Apparently, the ability to degrade 2-BE is strain-specific rather than species-specific.

To our knowledge, no strains of the genus *Hydrogenophaga*, *Cupriavidus*, or *Gordonia* have been described degrading EGEs like PEG or 2-BE. Nevertheless, other ethers such as AEs, aryl ethers, and alkyl aryl ethers are degraded by strains of these genera. For example, *Gordonia* spp. have been isolated growing on nonpolar 1,3- and 1,4-dialkoxybenzenes (Kim et al. 2007, 2008) or transforming ethyl *t*-butyl ether, MTBE, and *t*-amyl methyl ether most likely via the cytochrome P450 CYP249 yielding *t*-butyl alcohol or *t*-amyl alcohol, respectively (Malandain et al. 2010). *Hydrogenophaga flava* ENV735 is able to degrade MTBE (Hatzinger et al. 2001), and *Hydrogenophaga atypical* strain QY7-2 degrades 3-methyldiphenylether (Yang et al. 2016). *Cupriavidus* sp. WS degrades diphenyl ether, 4-bromodiphenyl ether, and 4,4′-bromodiphenyl ether (Wang et al. 2015). *Cupriavidus pinatubonensis* JMP134 (formerly *Ralstonia eutropha*, *Alcaligenes eutrophus*, and *Cupriavidus necator* JMP134) degrades 2,4-dichlorophenoxyacetic acid (2,4-D) via 2,4-D/α-ketoglutarate dioxygenase (TfdA) forming 2,4-dichlorophenol and glyoxylate (Don et al. 1985; Hogan et al. 2000). 2,4-D and 2-BAA are structurally similar; instead of the butyl moiety there is a chlorinated phenyl moiety. However, it is not known if strain JMP134 is able to degrade 2-BE or PEG.

Apart from BOE5, none of the isolated BOE strains could grow on any of the nonpolar ethers diethyl ether, di-*n*-butyl ether, *n*-butyl vinyl ether, and dibenzyl ether. For this reason, it is unlikely that 2-BE degradation is mechanistically similar to the degradation of nonpolar ethers. On the contrary, all isolated strains were able to grow on 2-BE, 2-BAA, *n*-butanol, and glyoxylic acid. 2-BAA, *n*-butanol, and butanoic acid were detected in a culture of BOE100 growing on 2-BE after the addition of Cm. These results lead to the assumption that the degradation of 2-BE by the isolated gram-negative strains proceeds via oxidation to 2-BAA and subsequent ether scission yielding butanol and glyoxylic acid, analogous to the degradation of PEG (Fig. 1, route 1).

The only known PEG degradation genes belong to strains *Sphingopyxis terrae* (GenBank accession no. AB239603), *Sphingopyxis macrogoltabida* strains 103 (AB196775) and 203 (AB239080), which share more than 99% sequence identity (Somyoonsap et al. 2008; Tani et al. 2007), and *Pseudonocardia* sp. K1 (AB126017) (Yamashita et al. 2004b). In the *Sphingopyxis* strains mentioned, PEG is oxidized by PEG dehydrogenase PegA and aldehyde dehydrogenase PegC to PEG carboxylate. PCDH catalyzes the ether cleavage forming glyoxylate and PEG shortened by one ethylene glycol unit. In strain *Pseudonocardia* sp. K1, the degradation of PEG proceeds in the same manner, but by different enzymes. The ether is cleaved by DGADH, which has a high homology with superoxide dismutase.

None of the PEG degradation genes *pegA*, *pegC*, *pcdh* or *dgadh* could be detected in the BOE strains by PCR (see Supplementary Material, supporting methods and Table S1). The primers used are, of course, very specific for the respective gene sequences. Therefore, it is still possible that the BOE strains use similar enzymes for the degradation of 2-BE. Further studies are necessary to identify these genes.

Degradation kinetics and 2-BE concentration limits were investigated for strains BOE3, BOE100, and BOE200. The maximum growth rate of BOE3 at 30 °C was 0.204 h^−1^, achieved at a concentration of 4 mM 2-BE. The degradation of 4 mM 2-BE took around 13 h. At higher concentrations, growth rates decreased continuously. The concentration limit for a steady degradation seemed to be 20 mM 2-BE which were consumed within 4 days. Concentrations higher than 20 mM led to substantially inhibited growth.

The maximum growth rate of BOE200 was 0.395 h^−1^ at 6 mM 2-BE which were degraded within 12 h. 20 mM 2-BE were removed within 3 days. Higher concentrations led to accumulation of 2-BAA and a pH drop to 6, and growth was strongly inhibited.

BOE100 grew the fastest and tolerated the highest substrate concentrations among the BOE strains. The maximum growth rate was 0.645 h^−1^ at a 2-BE concentration of 5 mM, which were completely degraded within less than 6 h. A stable degradation of 2-BE could be achieved for concentrations of up to 30 mM. Beyond that value, 2-BAA accumulated and the pH decreased to 6, inhibiting further growth, similar to BOE200.

These findings support the already stated hypothesis, that 2-BE is oxidized to 2-BAA before the ether bond is cleaved. In strains BOE100 and BOE200, the subsequent reaction seems to be the rate-limiting step, as 2-BAA was detected in the medium.

This study is the first that gives information about different 2-BE degrading bacterial strains and about degradation kinetics, although 2-BE is a HPVC and widespread in the environment. It is the main component of Corexit EC9527A, of which 813,000 L were applied to the ocean surface during the 2010 *Deepwater Horizon* oil spill. As a result, large quantities of 2-BE have been, and still are, released into the environment. Among the isolated strains, BOE100 degrades 2-BE the fastest and tolerates the highest concentrations. Moreover, it is able to degrade 2-BE in medium containing 3.5% NaCl corresponding to the salinity of seawater. Therefore, this strain is of great interest for a possible use in bioremediation.

Further studies are under way to identify the degradation genes and gain information about the enzyme kinetics.

## Electronic supplementary material

Below is the link to the electronic supplementary material.

Supplementary file1 (DOCX 14 kb)
