# Supplementary material for: Isolation and characterization of 2-butoxyethanol degrading bacterial strains
Source: Biodegradation. 2020 Apr 30;31(3):153–69. doi: 10.1007/s10532-020-09900-3 (PMC7299911; doi:10.1007/s10532-020-09900-3)
Supplement: Supplementary file 1 — Supplementary file1 (DOCX 14 kb) [file 10532_2020_9900_MOESM1_ESM.docx]

**Supplementary material**

**Isolation and characterization of 2-butoxyethanol degrading bacterial strains**

Christine Woiski*, Daniel Dobslaw, Karl-Heinrich Engesser

Department of Biological Waste Air Purification, Institute for Sanitary Engineering, Water Quality and Solid Waste Management, University of Stuttgart, Bandtaele 2, 70569 Stuttgart, Germany
* corresponding author, e-mail: christine.woiski@iswa.uni-stuttgart.de

**Supporting Methods**

PCR for the detection of PEG degradation genes

For the detection of the PEG degradation genes *pegA*, *pegC*, *pcdh*, and *dgadh*, PCR was used. DNA was isolated using GeneJET Genomic DNA Purification Kit (Thermo Fisher Scientific, Waltham, Massachusetts, USA). One PCR reaction mixture (total volume 25 µL) contained 2.5 µL buffer (10X), 2.5 mM MgCl_2_, 200 µM dNTPs, 0.625 U Taq polymerase (all Bio&Sell, Nuernberg, Germany), 200 nM of each primer (Microsynth Seqlab, Goettingen, Germany), 5 % dimethyl sulfoxide, and 1 µL DNA. Primers are listed in Table S1. PCR was performed under the following conditions: denaturation at 94 °C for 3 min; 30 cycles at 94 °C for 30 seconds, annealing at 51–57 °C (gradient) for 30 seconds, and elongation at 72 °C for 90 seconds; and a final elongation step at 72 °C for 5 min.

**Table S1** PCR primers

| Gene | Primer |  | GenBank accession no. |
| --- | --- | --- | --- |
| pegA | PegA-F | cccgcataactggagctt | AB196775 |
|  | PegA-R | gtccgtgttgatcgacca |  |
| pegC | PegC-F | ccactcacctcagacgtg | AB196775 |
|  | PegC-R | actggatgcgacctcaac |  |
| pcdh | Pcdh-F | gtcacggacggctttact | AB196775 |
|  | Pcdh-R | gttcaaggctcgtctcgt |  |
| dgadh | Dgadh-F | tgactatgatgaactagagcca | AB126017 |
|  | Dgadh-R | acaaattctgggcgacga |  |
